# Achieving Positive Mental Health and Wellbeing on the COVID-19 Frontline

**DOI:** 10.1016/j.xinn.2020.100024

**Published:** 2020-07-31

**Authors:** Vicky Poh Hoay Khoo, Julie Morsillo, Lei Zhang

**Affiliations:** 1 China-Australia Joint Research Centre for Infectious Diseases, School of Public Health, Xi'an Jiaotong University Health Science Centre, Xi'an, Shaanxi, China; 2 School of Arts and Social Sciences, Eastern College Australia, Melbourne, Australia; 3 Melbourne Sexual Health Centre, Alfred Health, Melbourne, Australia; 4 Central Clinical School, Faculty of Medicine, Monash University, Melbourne, Australia; 5 Department of Epidemiology and Biostatistics, College of Public Health, Zhengzhou University, Zhengzhou, Henan, China

## Main Text

Sweeping across the continents, the unprecedented Coronavirus crisis has transformed the images of health professionals in the world's eyes. Covered in personal protective equipment (PPE), faces barely recognized, these are the warriors who fight on the frontline for the health of the global community, and we, as part of the global community, in turn want to ensure that they too are well, both physically and mentally.

The pandemic is also unprecedented in the sense that it affects the world collectively. The shared experience provides the global community with a rare opportunity to learn from each other. Since China is the first country to combat the virus and has accumulated rich experiences in its prevention and control,^1^, ^2^, ^3^, ^4^, ^5^, ^6^ a mental wellbeing study of its frontline medical workers could be of significant value to the global community.

With this in mind, a qualitative study with semi-structured interviews was conducted in April 2020 with a medical assistance team recently returned from their frontline task in Wuhan, the epicenter of the outbreak in China. The interview questions were developed based on Marslow's hierarchy of needs, covering physiological needs, safety, love and belongings, emotional security, self-esteem, and self-actualization. It was intended to investigate if fulfilment of such needs helps promote mental wellbeing and vice versa. We understood that the team members had been filling out questionnaires on mental health throughout their stay in Wuhan and were not particularly keen to complete more paperwork. Therefore we opted to instead allow them to share their stories from their perspectives in their own words. As a result, the interviewees generally found the interviews cathartic and helpful in processing their experiences.

The team we interviewed comprised of 28 doctors and nurses. It was among the first batches of aid to arrive in Wuhan in late January. They subsequently stayed for two months. Before their arrival, Wuhan's medical staff had been fighting the outbreak for over a month. The sheer volume of patients and the dire shortages of medical equipment rendered them powerless, exhausted, and numb. The local health care system was on the verge of collapse.

Picking up the baton, the medical team, along with four other teams dispatched to Wuhan, admitted 110 (the full capacity of the modified hospital) of the accumulated 700 + patients awaiting treatment at the outpatient department within the first 3 days of their operation. Overshadowed by fear, the challenges they faced in the first 2 weeks were paramount:

- Much was unknown about the virus at the time. According to the interviewees, working closely with patients while not being fully aware of all modes of transmission was their greatest fear, followed by the infections and deaths of other medical staff.^7^
- The modified hospital was not equipped with the medical equipment they usually have access to at work, e.g., ventilators. They also had to conserve their PPE supply.
- Due to the delay in treatment, many patients were gravely ill when the medical team took over their care. There were many deaths in the first two weeks. The interviewees found that it was most distressing to lose their patients due to inadequate medical equipment, especially those in the prime of their lives.
- All interviewees expressed the uneasiness brought about by the eerie silence in the wards. Patients were passive and depressed, offering minimal communication. Despair and helplessness were pervasive.

**Figure 1:**
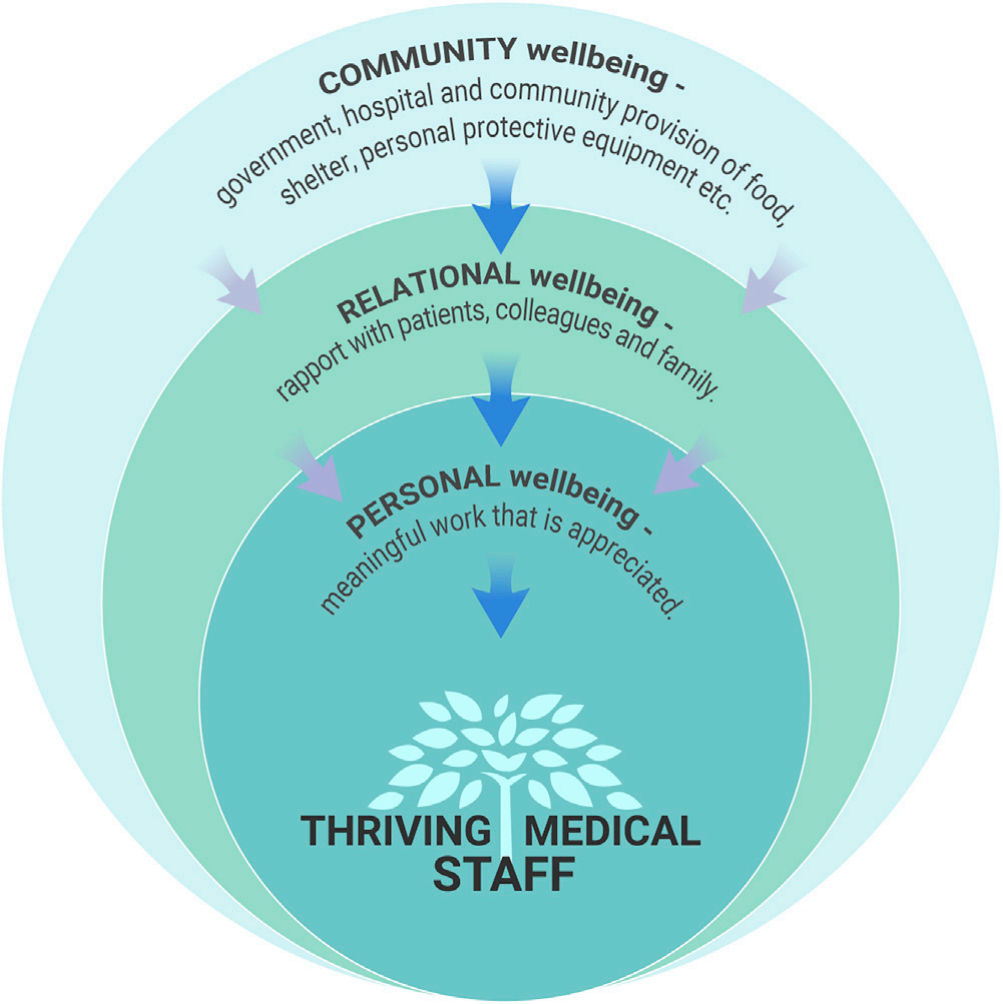
Achieving Positive Mental Health and Wellbeing on the COVID-19 Frontline

With what they had experienced on the frontline, one would expect them to be affected mentally and emotionally (the world has seen the mental toll of COVID-19 on health professionals, such as the tragic suicide of Dr. Lorna Breen).^8^ However, our research revealed a different picture. The interviews were conducted during their 14 days isolation period after they returned from Wuhan. One striking impression was the vibrancy of the team. They were energetic, positive, and motivated by new-found meaning in their lives and work. The following contributing factors were observed from the interviews (Figure 1).

### Meaningful Support from Government, Hospitals and Wider Community

The **basic needs** of the team members were thoroughly considered by the government, from the accommodation, food, rest to sports and leisure. Even the needs of their family members back in hometowns were well taken care of, with food and produce regularly delivered to their doorstep. Knowing this enabled the team to concentrate fully on their frontline tasks.

Their **safety** was ensured. Initially there were shortages of PPE, but supply was never cut off. Subsequent donations of PPE from the public and other countries also acted as protective factors for the team, both physically and mentally.

Local community support was presented in many forms during the team's stay in Wuhan, and best depicted by the ample donations of food and personal hygiene products. Some interviewees recalled the heart-warming act of the hotel management (their temporary accommodation in Wuhan), when upon learning that the team longed for their hometown food, prepared them the dishes despite difficulties in sourcing the ingredients in lockdown; the others mentioned how they ended up with stocks of personal hygiene products that could last them for months. All these gestures of appreciation conveyed the message that the team's efforts were valued and hence contributed to their mental wellbeing.

### Meaningful Support from Colleagues, Patients, and Families

The leaders set the tone of the team. Armed with 40 years of medical work experience and having previously participated in crisis missions, including the 2002 SARS and the 2008 Sichuan Earthquake, the chief leader was quick to establish work protocols and structure with the firm belief that that was the best tactic to help her team gain control and conquer the ubiquitous fear. With fierce determination, the five medical teams took over the operation of the hospital on the third day of their arrival. All leaders led their teams by example. They were the first to enter the isolation wards despite clutching fear.

The leaders went the extra mile for their members' wellbeing despite the scarcity of resources. Anxiety was building up after about a month because there was no prospect of returning home. In response, the leaders organized a mini-sports event. This initiative proved to be the game changer and sustained the teams for the rest of their stay in Wuhan.

There was a strong sense of comradeship among the team members. According to the interviewees, they kept details of the worst of their work from their families but were able to draw comfort from each other because of their shared understanding. At work, they helped each other out regardless of positions. A nurse recalled how a doctor came to her rescue when her protective eyewear fogged up and she was panicking from not being able to perform her task (for safety reasons, they were not allowed to touch their protective eyewear):

He said to me: ‘Let me be your eyes. Together we will prepare and administer the medications for the shift.’

The doctor-patient relationship in Wuhan was what the team could only dream of prior to the crisis. The doctor-patient relationship in China has been deteriorating in the past decades, with mutual distrust and violence against medical staff.^9^ Two major contributing factors to the doctor-patient rapport in Wuhan were that patients were appreciative and cooperative, knowing that the doctors and nurses risked their lives to help them; COVID-19 treatments were free to patients, a stark contrast to the exorbitant medical costs since the commodification of health care in China. Feeling that they were being valued and treated fairly, patients treated the doctors and nurses the same way.

Family members were supportive and took pride in the team members' decision to go to Wuhan. Most of the members maintained daily contact with their families. One head nurse recalled her husband saying:

If you fell sick, I would fight against all odds to come and look after you in Wuhan.

### New Meaningful Appreciation for Work and Life

Many of the interviewees made use of the 14 days isolation period to process thoughts and emotions. They did so through writing diaries, poems, or articles for newspapers; media interviews; and sharing with each other. Such reflection not only helped them to transition back to normal life but also enabled them to find new and meaningful perspectives to life.

The words of a nurse best illustrate this:

I used to regard my work in the ICU as repetitive and unmotivating, but now I am seeing it in a new light. I hold a special job, one that landed me in the unique position to offer warmth and care in a patient's last journey of life. This I wouldn't trade for the world.

Medical professionals are so good at caring for others' health, but often they overlook their mental wellbeing.^10^ The experience of the medical assistance team in Wuhan suggested that mental health care does not necessarily have to come from mental health professionals. Meaningful support could come from various sources. The best part is, we all can be part of the support network, be it being the empathetic colleague or the supportive family member or even the general public who is protecting the medical professionals by adhering to social distancing rules. We are all in this together, and together we will walk through this.
